# Supplementary material for: Casimir stabilization of fluctuating electronic nematic order
Source: Nat Commun. 2026 Jul 29;17:9211. doi: 10.1038/s41467-026-75828-6 (PMC13522547; doi:10.1038/s41467-026-75828-6)
Supplement: Supplementary file 1 [file 41467_2026_75828_MOESM1_ESM.pdf]

Supplementary Information: "Casimir Stabilization of Fluctuating Electronic Nematic Order"

Ola Carlsson,[1,2,*] Sambuddha Chattopadhyay,[1,3] Jonathan B. Curtis,[1] Frieder Lindel,[1,5] Lorenzo Graziotto,[4,5] Jérôme Faist,[4,5] and Eugene Demler[1,†]

[1]*Institute for Theoretical Physics, ETH Zürich, Zürich 8093, Switzerland*

[2]*Department of Physics and Arnold Sommerfeld Center for Theoretical Physics (ASC), Ludwig-Maximilians-Universität München, Theresienstr. 37, D-80333 München, Germany*

[3]*Lyman Laboratory, Department of Physics, Harvard University, Cambridge MA, USA*

[4]*Institute of Quantum Electronics, ETH Zürich, Zürich 8093, Switzerland*

[5]*Quantum Center, ETH Zürich, Zürich 8093, Switzerland*

REAL-FREQUENCY CASIMIR INTEGRAND

To gain further insights into the mode continuum origin of the Casimir control mechanism, we perform a spectral decomposition of the Casimir energy in Equation (1). As noted in Methods, this integration over imaginary frequencies $\omega = i\omega_m$ summarizes the contributions of photon modes of all real frequencies in a comparably small region on the imaginary axis. The relative importance of such real modes, with more direct physical interpretation than the imaginary frequency formulation, is elucidated through a contour integral manipulation which yields an alternate form of the Casimir free energy:

$$F(\theta) = -i\frac{\hbar}{2\pi}\int d\omega d\mathbf{k}_\perp \ln \det \left(1 - e^{2i\kappa_r d} R_e^{\alpha\beta}(\theta) R_b^{\beta\gamma}\right).$$

Here the frequencies ω are integrated over the positive real axis, inferring evaluation of the integrand at frequency argument $\omega + i\eta$ where η is an infinitesimal positive number. Note that this substitution should be carried out in all functions determining the reflection matrices $R_e(\theta)$, R_b —at the lowest level implying evaluation of the retarded linear response functions $\varepsilon_{\parallel/\perp}(\omega + i\eta)$ and $\sigma(\omega + i\eta)$— as well as substitutions of the form $\kappa \to -i\kappa_r = -i\sqrt{(\omega + i\eta)^2 - k_\perp^2}$.

We perform the integral over photon momentum azimuth ϕ, and plot the relative values of the Casimir integrand $f(\omega, k_\perp) = -i\int d\phi \ln \det \left(1 - e^{2i\kappa_r d} R_e^{\alpha\beta}(\theta) R_b^{\beta\gamma}\right)\Big|_{\theta=0}^{\theta=\pi/2}$, subtracting the free energies corresponding to the control energy scale ΔF, see Figure S1. We find that there are many dispersive modes contributing to the Casimir integrand. These include both traveling wave normal modes $\omega > k_\perp$ and modes evanescent between the planes $\omega < k_\perp$, where in addition the strong low frequency contributions differ in sign for the two cases. Each of these modes, however, are dispersive, i.e., are a 2D continuum of modes with different in-plane momenta. While it is indeed possible to identify contributions to the Casimir energy from specific dispersive modes in a given Casimir setup, such as from surface plasmon polaritons[1], these modes form already a mode continuum as a function of in-plane momentum.

This real frequency analysis also sheds some light on the practicality of the imaginary frequency formulation. Oscillating contributions from traveling waves here persist outside the region plotted. While the improper integral over such contributions require large integration domains, where finite integral domains can still be strongly cut-off dependent, this

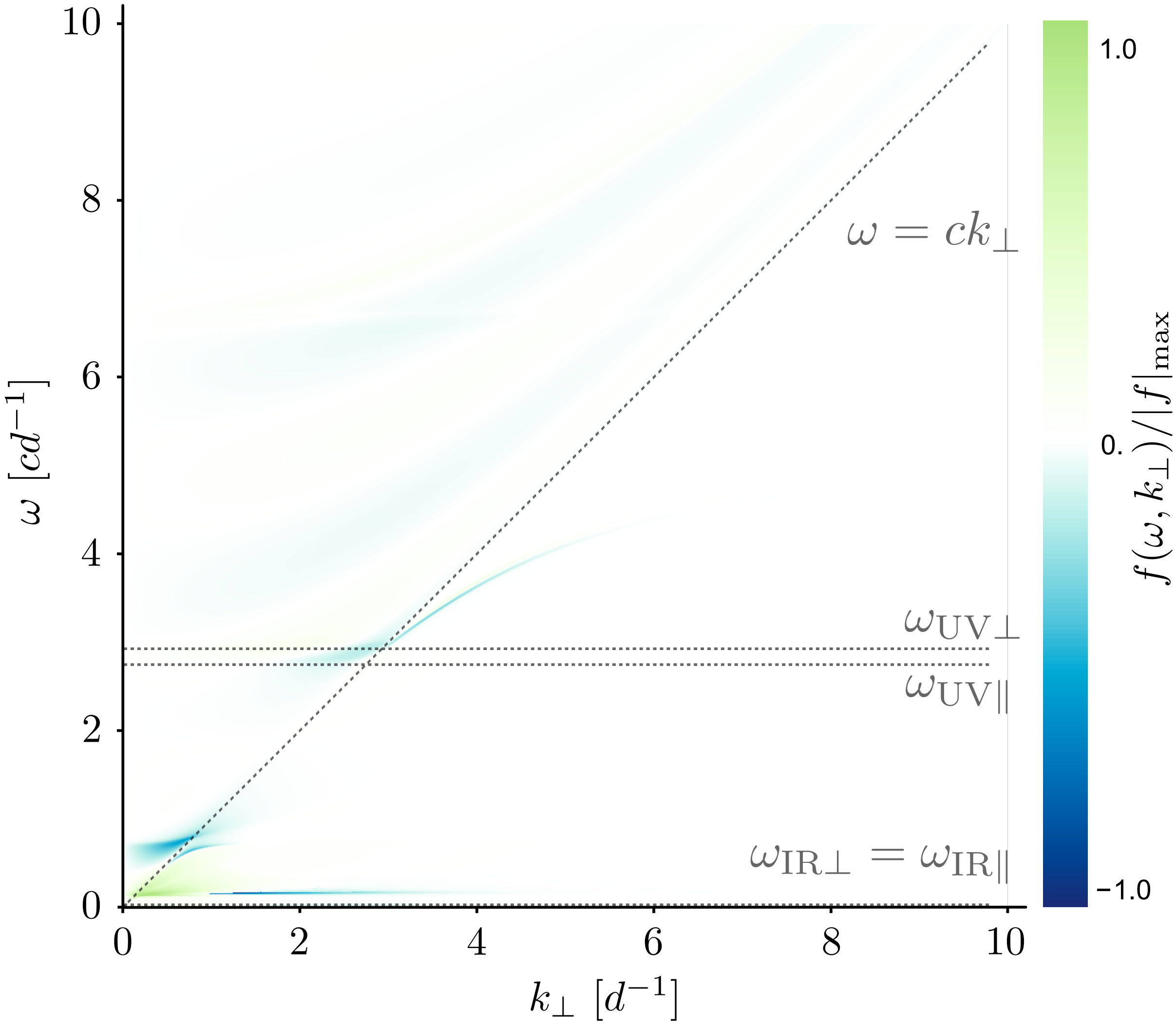

FIG. S1. *Normalized real-frequency resolved Casimir integrand* $f(\omega, k_\perp)$. The values corresponding to twist angle $\theta = \pi/2$ have been subtracted from the preferred $\theta = 0$. This illustrates the competing contributions from various normal modes of the system to the energy scale ΔF. Density is here $n = 2.9 \times 10^{11}$ cm^{-2}, Drude scattering time is $\tau = 1$ ns, 2DES anisotropy is $\lambda = 55$, distance is $d = 100$ nm, and 2DES filling factor is $\nu = 15/2$. Birefringent plate oscillator frequencies $\omega_{\mathrm{IR}\perp}$, $\omega_{\mathrm{IR}\parallel}$, $\omega_{\mathrm{UV}\perp}$, $\omega_{\mathrm{UV}\parallel}$, have been overlaid. The characteristic frequencies of the 2DES Ω_c, τ^{-1}, lie far below $\omega_{IR\perp} = \omega_{IR\parallel}$, and have not been overlaid. Contributions from the right hand side of the free photon dispersion $\omega = ck_\perp$ are due to modes evanescent between the planes, the left hand side of the same corresponds to traveling modes. Only dependence on photon transverse momentum magnitude $k_\perp$ is shown, and contributions from different azimuthal directions ϕ have been integrated over.

oscillatory behavior in ω implies exponential decay in the imaginary frequency $i\omega_m$, which is numerically much more stable.

Finally, the non-resonant nature of our control mechanism becomes apparent when overlaying the material resonance frequencies on our Casimir integrand. The most relevant frequencies are primarily set by the geometric length scale d of the problem, e.g. visible through the extent of the important low-frequency region stretching up to $\omega \sim c/d$. Clear features arising from the UV oscillator frequencies of the $BaTiO_3$ are also present, but far away from the natural frequencies τ^{-1}, Ω_c, of the electron system, which are orders of magnitude smaller and not marked out in Figure S1. It is rather the interplay of these vastly different resonant frequencies with the geometric frequency scale which control the Casimir mechanism, such that the photon modes that align nematic order are not close in energy to neither electronic modes nor optically active modes of the birefringent plate. Correspondingly, as seen in Fig. (3), the free energy shift is almost independent on the 2DES resonance frequencies Ω_c and τ^{-1}, as they lie far below the geometric frequency scale.

---

* ola.carlsson@lmu.de

† demlere@phys.ethz.ch

[1] Intravaia, F. & Lambrecht, A. Surface plasmon modes and the casimir energy. *Physical review letters* **94**, 110404 (2005).
