# Supplementary material for: Casimir stabilization of fluctuating electronic nematic order
Source: Nat Commun. 2026 Jul 29;17:9211. doi: 10.1038/s41467-026-75828-6 (PMC13522547; doi:10.1038/s41467-026-75828-6)
Supplement: Supplementary file 2 [file 41467_2026_75828_MOESM2_ESM.pdf]

nature portfolio

Peer Review File

Casimir Stabilization of Fluctuating Electronic Nematic Order

Corresponding Author: Mr Ola Carlsson

This file contains all reviewer reports in order by version, followed by all author rebuttals in order by version.

Attachments originally included by the reviewers as part of their assessment can be found at the end of this file.

Version 1:

Reviewer comments:

Reviewer #1

(Remarks to the Author)
The paper of Carlsson et al. is motivated within the field of Cavity Quantum Materials (Schlawin et al https://arxiv.org/abs/2112.15018, a review for reference). In fact, the authors (in a nutshell) define it in the first sentence of the abstract: “Vacuum cavity control of quantum materials …”. In this case, they investigate the nematic order of 2D materials when they are coupled to the enhanced zero-point energy of the electromagnetic field. In my opinion, the paper is interesting and of prominent quality. No doubt here.

However, my role here is that of a reviewer for a specific journal, and my comments should be understood in this context. Therefore, I suggest some observations that the authors may consider in a revised version of the manuscript.

The paper is motivated by the experiments (and theoretical analysis) of their Ref. 21. In fact, in some aspects Carlsson et al. can be understood as a follow-up to Graziotto et al., where many of the authors are shared between both publications. In addition, the authors cite a recent paper, their Ref. 16 (Kotov et al.), where a Casimir-like mechanism is discussed. Then:

i) In the discussion, the authors explain the differences between this new work and the previous one by Graziotto et al. In particular, they state that, in a cavity-QED system dominated by a main resonator mode, the setup considered in this work does not control the electron system. Again, in my opinion, this point must be further clarified. Specifically, the authors claim that the control is not a resonant process. This statement appears in several parts of the paper. Similar ideas (as far as I understand from reading Kotov et al.) have been discussed in Kotov et al., where they emphasize the importance of considering a continuum of modes.

ii) In a more technical discussion, the authors use the scattering “formalism,” whereas in Kotov a light–matter Hamiltonian perspective is adopted. I guess that both approaches should be equivalent. This is a sanity check and important for the community’s confidence. If so, I suggest checking some works where general formalisms for computing linear-response theory in light–matter systems are developed (also for multimode cases: Roman-Roche et al https://arxiv.org/abs/2406.11971). Among others, explicit formulas for the conductivities are given there, so some intuition might be gained by comparing resonant and continuum-like models.

iii) The paper is submitted to a generalist journal, so the above comments relating both approaches in cavity QED aim to contextualize the study for researchers working in this field. In this line, the authors could also comment on the differences between control of response functions and modification of the ground state of matter systems. This is related to the long history of the Dicke model and its generalizations. In the original work of Hepp and Lieb the equilibrium superradiant phase transition—or ground-state modification—although typically considered in a single-mode setting, is not a resonant phenomenon (multimode discussions are also available, e.g., Andolina et al https://arxiv.org/abs/2005.09088).

Therefore, the paper deserves to be published. It is a very good work. My comments are only intended to contextualize it and to suggest revisions in favor of readability and understanding for non-specialist readers of the chosen journal.

(Remarks on code availability)

Reviewer #2

(Remarks to the Author)
please see the review report in the attached file.

(Remarks on code availability)

Reviewer #3

(Remarks to the Author)
The manuscript presents a new approach to control quantum materials with fluctuating electronic nematic order by using the Casimir effect. As an example, the authors consider a two-dimensional electron system (2DES) in a perpendicular magnetic field at half-filled high Landau levels, where the system forms quantum Hall stripes. These stripes correspond to local domains with strongly anisotropic electrical conductance.

To control the orientation of the stripe domains, the authors propose to form a cavity between the 2DES and a strongly birefringent plate (BaTiO3). The resulting Casimir interaction favors an alignment of the conductivity easy axis of the Hall stripes with the optic axis of the birefringent plate. The authors refer to previous estimates showing that an energy of order 1 mK per stripe is sufficient to induce macroscopic ordering of the stripe orientation.

The main result of the paper is that the Casimir energy in the proposed setup is large enough for experimentally realistic parameters and can be up to four orders of magnitude larger than the required energy scale. In addition, the results depend only weakly on material parameters of the 2DES, such as electron density, filling factor, and scattering time.

Overall, the manuscript is well written, clearly structured, and the results are presented in a transparent and accessible way. The physical motivation is convincing, and the work is well embedded in the existing literature. In my view, the idea of using the Casimir effect to control the orientation of nanoscale domains in quantum matter is novel and very appealing.

However, regarding the validity of the presented results, I have some major concerns.

In principle, the methodology based on the scattering formalism used to compute the Casimir interaction energy is standard. Within this framework, the key ingredients are the reflection matrices of the two interacting surfaces. The authors employ well-known results for the birefringent plate, which is modeled as infinitely thick, following Refs. 32 and 51.

For the 2DES, the authors derive the reflection matrix using a phenomenological model for the conductivity tensor, combined with results from Ref. 52. While each of these ingredients may be reasonable when considered separately, the reflection matrices used for the two surfaces are not mutually compatible.

This incompatibility can be seen as follows. The reflection matrix of the birefringent plate is symmetric, which is a consequence of reciprocity when the polarization vectors defining the modes are orthonormal. For the 2DES, reciprocity is indeed broken due to the presence of the magnetic field. However, if one turns off the magnetic field and sets the Hall conductivity sigma_h to zero in the expressions following line 681, the resulting reflection matrix remains non-symmetric. This indicates that the polarization vectors underlying the definition of the reflection matrix for the 2DES are not orthonormal.

While either choice of polarization vectors is, in principle, acceptable when used consistently within the scattering formula (1), it is not correct to mix different conventions for the two reflection matrices. As a consequence, the numerical results presented in this work are likely not quantitatively correct, and I therefore suggest major revisions before making a final recommendation on the manuscript.

According to my own derivation, the off-diagonal elements of the reflection matrix following line 681 become compatible with the reflection matrix of the birefringent plate if one replaces omega_m^2 by omega_m*kappa in r_ps^e and kappa^2 by omega_m*kappa in r_sp^e (modulo missing factors of c, see minor comments).

Moreover, formula (1), which gives the Casimir energy per unit area for two parallel planar surfaces, is used here to estimate a Casimir energy per stripe (or per particle). However, it is well known that the Casimir interaction is generally non-additive. For this reason, the authors should be more critical about the applicability of this approximation and clearly state under which conditions it is expected to be valid.

In particular, the use of a planar energy density to estimate the energy of individual stripe domains is expected to be justified only when the lateral size of the stripe domains (typical values are not given in the manuscript) is large compared to the separation between the 2DES and the birefringent plate. In this regime, edge effects and non-additive contributions should be suppressed. Conversely, at larger separations, where the separation becomes comparable to or larger than the stripe domain size, the quantitative accuracy of the predicted energies is less clear. While the results at shorter distances may still be reliable, this limitation is not sufficiently discussed in the manuscript.

Minor comments:

- Line 198: The quantities appearing in the expression for the filling factor (such as n and B) are not explicitly defined,

although their meaning is rather standard. For clarity and completeness, these quantities should be briefly explained when they first appear.

- Line 651: While most quantities entering the subsequent equation are reintroduced and explained, the anisotropy ratio \lambda is not defined again at this point. I suggest to restate its definition here for completeness and readability.

- In several parts of the Methods section, the speed of light c appears to be set to unity, while in the main text it is not. This can be seen, for example, when comparing the expression for \kappa following Eq. (1) with the expressions for the reflection matrix following line 628. See also the formulas following line 674.

(Remarks on code availability)

Version 2:

Reviewer comments:

Reviewer #2

(Remarks to the Author)
Thank you for the detailed responses. All of my concerns have been addressed.

(Remarks on code availability)

Reviewer #3

(Remarks to the Author)
Dear editor, dear authors,

I would like to thank the authors for their detailed responses and the substantial effort put into revising the manuscript. They have addressed the vast majority of my previous concerns effectively, and I believe the manuscript is significantly stronger as a result.

I have only a few remaining minor remarks and clarifications before recommending the manuscript for publication:

1. There are a couple of minor inconsistencies with equation citations. Following Equation (1), the text cites "Eq. (4)", which likely should refer to Equation (1) itself, as no Equation (4) exists in the manuscript. Equation (4) is also referenced in the paragraph before the methods section, in the first paragraph of the methods section, and right before the final form. Please double-check and correct these references.

2. There appears to be a minor dimensional issue regarding the reflection matrix for the 2DES. While the conductivity $\simga$ seems to be defined in SI units, the current expression for the reflection coefficient seems to assume it is dimensionless. It looks like the vacuum impedance (Z_0) might be missing from the reflection formula. Furthermore, to avoid any ambiguity, it would be worthwhile to explicitly state in the methods section that the SI unit system is chosen.

Once these final minor points are integrated, I am fully in support of the publication of this work.

(Remarks on code availability)

Open Access This Peer Review File is licensed under a Creative Commons Attribution 4.0 International License, which permits use, sharing, adaptation, distribution and reproduction in any medium or format, as long as you give appropriate credit to the original author(s) and the source, provide a link to the Creative Commons license, and indicate if changes were made.
In cases where reviewers are anonymous, credit should be given to 'Anonymous Referee' and the source.
The images or other third party material in this Peer Review File are included in the article's Creative Commons license, unless indicated otherwise in a credit line to the material. If material is not included in the article's Creative Commons license and your intended use is not permitted by statutory regulation or exceeds the permitted use, you will need to obtain permission directly from the copyright holder.
To view a copy of this license, visit https://creativecommons.org/licenses/by/4.0/

May 15, 2026

Dear Referees,

We are delighted to return to you a revised version of our manuscript "Casimir Stabilization of Fluctuating Electronic Nematic Order" as well as a reply to all of your comments. We would like to thank you for the thorough reading of our manuscript and for your time and effort in reviewing our manuscript. We have significantly modified the manuscript based on your feedback. Additionally, we thank for the positive feedback received, and are glad to see interest in our work. If not otherwise specified, references to equations, literature, and figures are indexed by their appearance in the revised Manuscript.

Yours,

Ola Carlsson, Sabuddha Chattopadhyay, Jonathan B. Curtis, Frieder Lindel, Lorenzo Graziotto, Jérôme Faist, and Eugene Demler.

In the following we address the comments and concerns raised by each Referee in turn, and give a full list of changes made in the manuscript.

Referee # 1

> The paper of Carlsson et al. is motivated within the field of Cavity Quantum Materials (Schlawin et al https://arxiv.org/abs/2112.15018, a review for reference). In fact, the authors (in a nutshell) define it in the first sentence of the abstract: "Vacuum cavity control of quantum materials ...". In this case, they investigate the nematic order of 2D materials when they are coupled to the enhanced zero-point energy of the electromagnetic field. In my opinion, the paper is interesting and of prominent quality. No doubt here.
>
> However, my role here is that of a reviewer for a specific journal, and my comments should be understood in this context. Therefore, I suggest some observations that the authors may consider in a revised version of the manuscript.

We are happy to hear that the paper appears of interest, and we will leverage the supplied comments into further improvement.

> The paper is motivated by the experiments (and theoretical analysis) of their Ref. 21. In fact, in some aspects Carlsson et al. can be understood as a follow-up to Graziotto et al., where many of the authors are shared between both publications. In addition, the authors cite a recent paper, their Ref. 16 (Kotov et al.), where a Casimir-like mechanism is discussed. Then:
>
> i) In the discussion, the authors explain the differences between this new work and the previous one by Graziotto et al. In particular, they state that, in a cavity-QED system dominated by a main resonator mode, the setup considered in this work does not control the electron system. Again, in my opinion, this point must be further clarified. Specifically, the authors claim that the control is not a resonant process. This statement appears in several parts of the paper. Similar ideas (as far as I understand from reading Kotov et al.) have been discussed in Kotov et al., where they emphasize the importance of considering a continuum of modes.

That the Casimir control mechanism proposed in the Manuscript is distinct from a few-mode cavity QED approach is indeed an important point of this paper. That Casimir control mechanism depends on the full continuum of electromagnetic field modes is crucial, and we have emphasized this further in several of the added comments, see below. While an analysis based on a fundamental mode only is not necessarily applicable to our considered experimental setup, we may still apply the Casimir control formalism to setups such that of Graziotto et al. The comments on lines 558 and following serve rather to highlight the distinction between the setup considered in this work and the one implemented by Graziotto et al. Casimir energies can also be computed in general resonator configurations, as pointed out in the same paragraph 558-585, as well as the new Ref. [65].

In particular, we have performed a spectral decomposition of the Casimir free energy, i.e., an evaluation of the Casimir integrand [Manuscript Equation (1)] expressed in terms of *real* frequencies ω, see Appendix of the revised manuscript. For clarity this plot is also reproduced here in Reply Figure 1. For example, at fixed ω, this plot shows the free energy difference due to the different in-plane momenta of the field in the setup at that frequency. A few features are apparent which should clarify our claim of mode continuum importance:

1. We see in Fig. 1 that there are many dispersive modes contributing to the Casimir integrand. These include both traveling wave normal modes $\omega > k_\perp$ and modes evanescent between the planes $\omega < k_\perp$, where in addition the strong low frequency contributions

differ in sign for the two cases. Each of these modes, however, are dispersive, i.e., are a 2D continuum of modes with different in-plane momenta. Moreover, they are smeared out in frequency, such that one can also not assign a single frequency to them at a fixed momentum. So while it is indeed possible to identify contributions to the Casimir energy from specific dispersive modes in a given Casimir setup, such as from surface plasmon polaritons ([2], this reply), these modes form already a mode continuum as a function of in-plane momentum.

2. Moreover, we still observe oscillating contributions from traveling modes for a large extent of higher frequencies ω, past the range plotted in Figure 1. This illustrates not only the difficulties of analyzing single modes (a subset of oscillating contributions can sum to something very different from the improper integral), but also stresses the necessity of the imaginary (Matsubara) frequency formulation used in the manuscript to find convergence of the integral, see also Ref. [35]. Shifting the integral to imaginary frequencies, oscillations in ω turn into exponential decay in $i\omega_m$, bringing vastly improved numerical stability.

3. Overlaying the material frequencies ω_{IR}, ω_{UV}, Ω_c, and τ^{-1} on the "spectral decomposition" shows how the dominant frequencies are not set by resonances of the 2DES or birefringent plate (whose role would be filled by a separate cavity in more traditional cQED setups), but rather by an interplay between these resonant frequencies, and most importantly the geometric frequency scale set by the interplane distance d. That the Casimir control mechanism is not resonant is here abundantly clear: The photon modes that align electron nematic order are not of comparable frequency to neither electronic modes nor optically active modes of the birefringent plate. Correspondingly, as seen in Fig. (3), the free energy shift is almost independent on the 2DES resonance frequencies Ω_c and τ^{-1}.

ii) In a more technical discussion, the authors use the scattering "formalism," whereas in Kotov a light–matter Hamiltonian perspective is adopted. I guess that both approaches should be equivalent. This is a sanity check and important for the community's confidence. If so, I suggest checking some works where general formalisms for computing linear-response theory in light–matter systems are developed (also for multimode cases: Roman-Roche et al https://arxiv.org/abs/2406.11971). Among others, explicit formulas for the conductivities are given there, so some intuition might be gained by comparing resonant and continuum-like models.

While a light-matter Hamiltonian perspective should indeed agree with the scattering formulation if sufficient care is taken, such a direct comparison is complicated by a few factors. Most prominently, as discussed in Kotov et al. (our Ref. [35], new Manuscript), direct summation of the zero point energy of a Hopfield-like Hamiltonian is divergent. This divergence arises from the infinite zero point energy of the vacuum EM field, which can be consistently subtracted away (as shown by Barash and Ginzburg) leading to the Casimir-Lifshitz calculations employed in our manuscript, see also the discussion of comment # 8 of Referee # 2. While polariton modes in e.g. a Fabry-Pérot cavity can be indexed by a discrete variable (a in Kotov) for each transverse momentum, the vacuum EM background has continuous contributions which need then be subtracted from this discrete sum. This is precisely what happens e.g. when using the Euler-Maclaurin summation rule for the Casimir effect between two perfect conductors, which would have to be extended to the interacting polariton case. Indeed Ref. 35 finds the scattering formalism to be the well-behaved approach to this class of light-matter interacting systems, where the connection between the Hopfield-like Hamiltonian and Casimir-Lifshitz theory is made explicit through calculations following Barash and Ginzburg, see SI II of Ref. [35]. More direct applications of few-mode light-matter Hamiltonians, such as the Hopfield-like Hamiltonian, do indeed not agree with the renormalized Casimir result, and are therefore ill-behaved, as found by Kotov et al. [35].

> iii) The paper is submitted to a generalist journal, so the above comments relating both approaches in cavity QED aim to contextualize the study for researchers working in this field. In this line, the authors could also comment on the differences between control of response functions and modification of the ground state of matter systems. This is related to the long history of the Dicke model and its generalizations. In the original work of Hepp and Lieb the equilibrium superradiant phase transition—or ground-state modification—although typically considered in a single-mode setting, is not a resonant phenomenon (multimode discussions are also available, e.g., Andolina et al https://arxiv.org/abs/2005.09088).
>
> Therefore, the paper deserves to be published. It is a very good work. My comments are only intended to contextualize it and to suggest revisions in favor of readability and understanding for non-specialist readers of the chosen journal.

The dichotomy between response function modification (which includes regimes of driven cavity fields) and ground state manipulation is worthwhile to point out. Indeed the superradiant phase of the Dicke model, despite the original formulation's inherent problems with a spatially homogeneous single-mode EM field, is a paradigmatic off-resonant ground state modification, with the contrast between off-resonant ground state modification towards more traditional resonant modification in *driven* systems already pointed out by Hepp & Lieb. As of such, we add a comment to this effect in line 65 of the revised manuscript.

Referee # 2

> The theoretical manuscript proposes controlling the orientation of fluctuating nematic order in the quantum Hall stripe phase of a 2D electron system (2DES) by placing it near a birefringent BaTiO3 plate and exploiting an orientation-dependent Casimir (vacuum) free energy. The Casimir free-energy contribution is computed within a scattering-formalism framework from Ref. [35] and [36] , using reflection matrices constructed from a dielectric tensor for BaTiO3 and a modified Drude-type conductivity tensor for the anisotropic (stripe) 2DES. The manuscript then studies how the resulting stabilization energy varies with twist angle and with the separation between the 2DES and the birefringent plate, and argues that this energy scale can align otherwise fluctuating stripe domains, yielding a preferred macroscopic nematic orientation. The central idea is interesting, and the calculations appear internally consistent within the stated model; however, several key claims (especially regarding experimental robustness, physical interpretation, and positioning in the broader cavity–vacuum-control literature) require clearer justification and/or additional analysis before the conclusions can be considered convincing.

We appreciate the interest in the proposed control mechanism, and hope that our revision can lay doubts regarding our conclusions to rest.

> Major comments Comment # 1: The manuscript would benefit from a clearer statement of the main conceptual/technical contribution. The scattering-theory approach to Casimir free energy is not new (Ref. [35] and [36]), and the present work appears to apply a known formalism by inserting specific material response functions (BaTiO3 dielectric tensor and stripe-state conductivity) to obtain an angle-dependent free energy; the authors should articulate explicitly what is new beyond this application (e.g., a new mechanism, a new scaling regime, or a new experimentally relevant estimate).

Indeed, we have found that well-known methods from the Casimir community, and in particular discussions of the Casimir torque, can be directly used for a robust description of

the control mechanism we set out to suggest. From a technical point of view, we incorporated the scattering at the quantum Hall stripe system into the Casimir framework, by computing the reflection matrices of a quantum Hall stripe system.

Our main contribution in this regard is of less technical nature and lies in connecting results found in the field of dispersion forces (Casimir physics) with ongoing efforts of vacuum cavity engineering of condensed matter systems, where Casimir control schemes of this type have, to the best of our knowledge, not yet been applied. In addition to this, the favorable magnitude of the Casimir energy scale we find (i.e. numerical results) for this setup constitute a novel indication that our proposed mechanism is experimentally relevant. The discussion on lines 71-75 has been adapted to state our own contributions more clearly.

> Comment # 2: The physical mechanism is presented mainly in terms of the scattering formula for the free energy, but the manuscript does not clearly explain how the electromagnetic normal modes (or, equivalently, the photonic density of states) change with twist angle and how that translates into the observed angular dependence of the free energy. A qualitative explanation (even schematic) connecting twist angle, mode structure, to free-energy anisotropy would greatly improve the manuscript.

We agree that a qualitative discussion on the structure of the EM normal modes, and their subsequent twist-angle dependent free energy, would bring much welcome physical intuition to the effect. Unfortunately, the mode structures in the presence of birefringent media are involved to the point of intractability, see Figure 1 of this reply and its discussion. However, the preferred alignment of optic axes is a well-known result in the Casimir torque literature and can be understood schematically:

Consider to this effect an idealized birefringent material where reflection along the optic axis is perfect, while the dielectric function in other directions is that of vacuum, i.e. the material is transparent in the orthogonal direction. If two slabs of this material are placed in planar configuration with their optic axes aligned, photons polarized along the shared optic axis see Dirichlet boundary conditions, effecting the prototypical Casimir scenario for that polarization, while remaining polarizations are simply free space photon modes. Conversely, if the optic axes are orthogonal to one another, polarizations along either optic axis always see one slab as transparent, and there is no geometry dependent Casimir energy. The first configuration is therefore the only one with finite and negative Casimir energy and thus preferred over the second configuration with orthogonal optic axes. We have not included this discussion in the manuscript, as the alignment of optic axes is well-known in the Casimir community, and previously well studied, as we point out in lines 399-404.

> Comment # 3: It is unclear whether (and how) dielectric screening and electrostatics associated with the nearby plate/substrate are treated consistently. In a real device, a nearby dielectric modifies Coulomb interactions in the 2DES and can shift energetics of stripe formation itself; the manuscript should clarify whether the “control plate” is assumed to only affect the electromagnetic vacuum fluctuations or also modifies electron–electron interactions through static/dynamic screening, and what the expected magnitude of that effect is.

Our work does not consider any modification from the nearby dielectric on the energetics of stripe formation or on the microscopics of the electronic system, but only on the stabilization of fluctuating pre-formed domains. In that, it accounts for the full electromagnetic continuum including longitudinal quasistatic and static contributions.

Static screening of the birefringent plate will also affect the Coulomb interaction between electrons, however only at length scales larger than the distance between the planes d. Moreover, Hartree-Fock energetics of stripes are sensitive to the Coulomb interaction at length scales *smaller* than the cyclotron radius R_c (discussed in the response to Ref. # 3), for our system $\sim 30 - 50$ nm, it would not be surprising if electrostatics indeed do

change the energy landscape of stripe formation at the smaller distances we identify in our "Casimir control window". Two arguments could be made regarding such a modification: First, Hartree-Fock energies of stripe formation are large compared to typical temperatures considered, at a scale of single Kelvins per particle. Experimental observation of stripes happen first in the ~ 100 mK regime, a deviation usually ascribed to precisely the fluctuating domains we aim to stabilize. This means that even a considerable change of $\sim 10\%$ to stripe formation energies might not affect observation, as stripes remain disordered at temperatures still far below this modified critical temperature. Second, although more complicated to treat theoretically, significant electrostatic impact at smaller distances indeed constitute additional, richer, vacuum cavity modifications of a strongly correlated electron system. If it is the case that stripe formation is severely impacted at small < 50 nm distances, this grants the possibility for experiments to observe new physics of solid interest of its own, in the same setup that would display stripe stabilization at larger distances where electrostatics do not matter. In the revised manuscript we comment on these considerations on lines 248-253 and 728-752.

> Comment # 4: Figure 3 reports a stabilization energy vs separation d and implicitly implies strong distance dependence. If the Casimir free energy changes significantly with d, there is also an associated normal Casimir force between the bodies; the manuscript should estimate the magnitude of this force (or force per area) for the parameter regime used and comment on whether such forces are mechanically reasonable/compatible with typical experimental constraints, ideally with at least one comparison to measured Casimir-force scales in related geometries.

Measuring Casimir forces (pressures) typically require exquisite sensitivity due to the relatively small magnitudes involved. As an example, the pressure in the first precision measurement of the Casimir force in the parallel plate geometry ([1], this reply) was on the order of 10^{-3} Pa, where it was measured through shifts of a cantilever frequency. For setups not designed to be sensitive to these pressures, we do not expect apparatus control to be hampered by such small forces. We have calculated an upper bound on the Casimir pressures involved in our present geometry, which follows from considering retarded $F \sim d^{-3}$ behavior even though the true exponent is smaller in our typical distance regime. These estimates show the Casimir pressure to lie below 0.14 Pa and 400 Pa for 100 and 10 nm inter-plane distance respectively. This should be compared with the corresponding pressures of ~ 13 and $\sim 1.3 \times 10^5$ Pa for the same distances in the case of perfectly reflecting metal surfaces, showcasing the strong reduction of Casimir pressures in our setup due to reduced reflection. Being far below even the perfect conductor Casimir force values, it is hard to imagine the Casimir pressures having any noticeable impact on experiment.

Note that the smaller distance regime towards ~ 10 nm is most likely only accessible when controlling the distance through a spacer layer placed between the birefringent plate and the semiconductor heterostructure hosting the quantum Hall systems. As of such, the Casimir pressure should be compared to the compressive strength of the spacer in this regime. Unsurprisingly, as this lies (far) above the MPa range for most materials, even in the small distance limit we do not expect finite Casimir forces to complicate realization.

> Comment # 5: The manuscript emphasizes that single/few-mode cavity-QED pictures are insufficient and that continuum-mode Casimir physics is essential. However, no spectral decomposition is provided to show which frequency and in-plane momentum ranges dominate the stabilization energy (or the difference in free energy between orientations), so the continuum nature remains asserted rather than demonstrated. It would be nice to add quantitative analysis/comparison to clarify this point.

We agree with the Referee. Through a spectral decomposition in real frequency of the Casimir energy reported in Figure 1 of this reply, we hope to demonstrate this point more

convincingly. This decomposition has also been added to the Manuscript in the Appendix. See the discussion in the reply to Referee 1 and the Appendix for further comments on this spectrum.

> Comment # 6: The introduction states broadly that vacuum-field cavity control has been explored across ferroelectricity, superconductivity, and ferromagnetism, but several important and widely cited contributions from other groups are missing. At minimum, the literature overview should be expanded so that the manuscript does not read selective; the authors should cite representative works from multiple communities (electron–photon coupling in solids, phonon-polaritonic/ferroelectric routes, cavity-modified superconductivity, cavity-magnetism proposals), and clarify how their "Casimir control" differs from those approaches.

We have added a further references to previous theoretical works on vacuum-field cavity control, as well as the experimental results on YCBO superconductivity and magnetism. These amount to References [7-8], [11-15], [17-29] in the revised Manuscript. The cited literature is now hopefully more representative of the current state of the field.

> Comment # 7: Relatedly, additional review articles on cavity control of materials (beyond the single review currently cited) should be included, and the introduction should be revised to better reflect the breadth of the field and avoid giving the impression that only a narrow set of early proposals exist. The manuscript would benefit from a short paragraph that explicitly distinguishes "Casimir control" (renormalized, mode-continuum, non-resonant, geometry/material engineered) from more standard cavity-QED-inspired approaches.

An additional review, Ref. [4] in the revised Manuscript, is now included in the introduction reviews to further widen the breadth of literature cited.

> Comment # 8: The manuscript's framing would be strengthened by engaging with recent work in Fabry–Pérot-like cavity settings showing that, in some regimes, a continuum of photon modes can be approximated by one or a few effective modes while still removing free-space contributions through appropriate renormalization. This is directly relevant to the manuscript's claim that single/few-mode descriptions are generically insufficient, and the authors should clarify the scope/limits of that claim.

We thank the Referee for pointing this out. We have made brief comments on lines 604-615 on that careful treatments of singled-out photonic modes might also resolve issues pertaining to the free-space photon background. In particular the new Ref. [69] constitutes such a recent effort towards handling mode continuum and renormalization concerns within an effective few-mode theory. This work further highlights the importance of considering the free photon contribution to physical properties, and shows a trajectory towards incorporating this within existing cavity QED frameworks under certain approximations. It is also noteworthy that the zero point energy of infinite mode cavity QED formulations can be manipulated into direct correspondence with the Casimir free energy, see comments to Referee # 1.

> Comment # 9: The key quantitative claim is that the orientation-dependent Casimir free energy can reach "stabilization energy scales up to 10^4 times larger than previously known mechanisms" for stripe alignment. This claim is extremely strong, yet the manuscript does not provide a sufficiently careful uncertainty analysis (dielectric function model choices, heterostructure layering, losses, finite thickness) to justify it as a general or experimentally robust statement.

Indeed the estimates presented in this work are not a full simulation of experimental conditions. We have ensured the stability of the energy against variations of $BaTiO_3$ dielectric function parameters, and note that the employed two-oscillator model has seen good agreement with experimental data for e.g. the measurement of the Casimir torque, Ref. [52] in the revised Manuscript. The 2DES conductivity model is most probably the largest source of uncertainty, where no explicit theory or measurement of the dynamical behavior of stripe longitudinal resistivity exists. The take-home message of Manuscript Fig. 3 (b) however, is that variations of dynamical parameters within the Drude model have an astonishingly small impact on Casimir energy scales, a reflection of the highly non-resonant nature of our mechanism. Finally, the impact of finite thickness is natively expected to be small in our setup. The relevant length scale for Casimir physics is the interplane distance d, which sets the relevant wave lengths and the critical thickness at which the modeling of the slab as an infinite half-plane breaks down (see e.g. Ref. [52], revised Manuscript or [3]). As we consider distances in the nanometers, we expect the finite thickness of the birefringent slab to have negligible impact on the setup. Conversely, the modeling of the electron system as two-dimensional hinges on the width of the quantum well being smaller than the interplane distance. Typical GaAs wells are of the order ~ 5 nm, motivating this approximation well for all but the smallest distances considered. We have added comments on this matter at lines 517-531.

> Minor comments
> Comment # 10: When introducing Eq. (1) (the scattering-formalism expression for the free energy), it would be helpful to briefly state the key assumptions and limitations (e.g., planar geometry, linear response, locality/nonlocality assumptions, how material dispersion and losses enter, and what is meant by the renormalization/subtraction).

We have added some additional clarification on the assumptions behind Equation (1), on lines 306-323.

> Comment # 11: In Fig. 3, the manuscript should explicitly state which twist-angle configuration is used to define the plotted stabilization energy, so that the plotted quantity is unambiguous.

We thank the Referee for pointing out this detail. The precise angle $\theta = \pi/2$ is now clearly stated on lines 418-419 and in the caption of Manuscript Figure 3.

> Comment # 12: Please justify (briefly) the specific parameter choices used in the main figures (e.g., d, nu, tau, and n) and clarify whether they are representative of particular experiments or chosen for numerical convenience.

Indeed the chosen 2DES parameters have been estimated from typical GaAs values, more precisely from Ref. 62, revised Manuscript, while the initial distance of 100 nm is simply a small distance still comfortably within experimental considerations. This has been clarified on lines 396-399. Nevertheless, the impact of these choices are all illustrated in Figure 3, where in particular the estimate of the 2DES parameters is found to be of minor importance.

> Comment # 13: On page 6, the authors mentioned that "Indeed, by considering this geometry, we broaden the scope of electromagnetic vacuum control outside of the commonly considered vacuum cavity regime." It seems that the control is still within the scope of cavity control, if we define a cavity as a device that confines electromagnetic vacuum within a confined space such as plaritonic devices and hBN phonon-polaritonic devices.

We agree fully that this sentence may be strongly worded depending on what working convention of "cavity" is adopted. As written, it considered a cavity as an object with discrete resonances. The sentence has been removed in the updated Manuscript.

> Comment # 14: The acronym "2DES" appears before being defined (it is used on page 1 but defined later); please define it at first use for readability.

This has been corrected. We thank the Referee for pointing this out.

Referee # 3

> The manuscript presents a new approach to control quantum materials with fluctuating electronic nematic order by using the Casimir effect. As an example, the authors consider a two-dimensional electron system (2DES) in a perpendicular magnetic field at half-filled high Landau levels, where the system forms quantum Hall stripes. These stripes correspond to local domains with strongly anisotropic electrical conductance.
>
> To control the orientation of the stripe domains, the authors propose to form a cavity between the 2DES and a strongly birefringent plate (BaTiO3). The resulting Casimir interaction favors an alignment of the conductivity easy axis of the Hall stripes with the optic axis of the birefringent plate. The authors refer to previous estimates showing that an energy of order 1 mK per stripe is sufficient to induce macroscopic ordering of the stripe orientation.
>
> The main result of the paper is that the Casimir energy in the proposed setup is large enough for experimentally realistic parameters and can be up to four orders of magnitude larger than the required energy scale. In addition, the results depend only weakly on material parameters of the 2DES, such as electron density, filling factor, and scattering time.
>
> Overall, the manuscript is well written, clearly structured, and the results are presented in a transparent and accessible way. The physical motivation is convincing, and the work is well embedded in the existing literature. In my view, the idea of using the Casimir effect to control the orientation of nanoscale domains in quantum matter is novel and very appealing.

We are happy to hear that the Referee finds the work of interest and the underlying idea appealing.

> However, regarding the validity of the presented results, I have some major concerns.
>
> In principle, the methodology based on the scattering formalism used to compute the Casimir interaction energy is standard. Within this framework, the key ingredients are the reflection matrices of the two interacting surfaces. The authors employ well-known results for the birefringent plate, which is modeled as infinitely thick, following Refs. 32 and 51.
>
> For the 2DES, the authors derive the reflection matrix using a phenomenological model for the conductivity tensor, combined with results from Ref. 52. While each of these ingredients may be reasonable when considered separately, the reflection matrices used for the two surfaces are not mutually compatible.
>
> This incompatibility can be seen as follows. The reflection matrix of the birefringent plate is symmetric, which is a consequence of reciprocity when the polarization vectors defining the modes are orthonormal. For the 2DES, reciprocity is indeed broken due to the presence of the magnetic field. However, if one turns off the magnetic field and sets the Hall conductivity σ_h to zero in the expressions following line 681, the resulting reflection matrix remains non-symmetric. This indicates that the polarization vectors underlying the definition of the reflection matrix for the 2DES are not orthonormal.

> While either choice of polarization vectors is, in principle, acceptable when used consistently within the scattering formula (1), it is not correct to mix different conventions for the two reflection matrices. As a consequence, the numerical results presented in this work are likely not quantitatively correct, and I therefore suggest major revisions before making a final recommendation on the manuscript.
>
> According to my own derivation, the off-diagonal elements of the reflection matrix following line 681 become compatible with the reflection matrix of the birefringent plate if one replaces ω_m^2 by $\omega_m\kappa$ in r^e_{ps} and κ^2 by $\omega_m\kappa$ in r^e_{sp} (modulo missing factors of c, see minor comments).

The Referee points out that two different normalizations of polarization vectors had been used for the derivations of the reflection coefficients. On closer examination, we agree completely on the presence and nature of this issue, and apologize that such an error made it into the final manuscript. Indeed the polarization vectors used in the derivation of the birefringent plate reflection coefficients, found in Ref. [76], revised Manuscript, read in our notation:

$$\Pi_s = \begin{pmatrix}0\\1\\0\end{pmatrix}$$
$$\Pi_p = \begin{pmatrix}\kappa c/\omega_m\\0\\\pm ikc/\omega_m\end{pmatrix},$$

with $\pm$ denoting upwards and downwards propagating waves, respectively. Equation (3) instead gives the reflection coefficients as a function of electric field polarization directions in the x-y plane, i.e. for the polarization vectors

$$\Pi'_s = \begin{pmatrix}0\\1\\0\end{pmatrix}$$
$$\Pi'_p = \begin{pmatrix}1\\0\\\pm ik/\kappa\end{pmatrix}.$$

This mismatch lead to inconsistent reflection coefficients r_{sp}, as pointed out by the Referee. Performing a basis transformation of the reflection matrix $R_e^{\alpha\beta}$ into the appropriate in-plane polarization basis $\begin{pmatrix}\kappa c/\omega_m\\0\end{pmatrix}$, $\begin{pmatrix}0\\1\end{pmatrix}$ results in the corrected 2DES reflection coefficients:

$$\begin{aligned}
r^e_{ss} &= -g^{-1}(\sigma_{ss}(2\omega_m^2/c^2 + \kappa\omega_m\sigma_{pp}/c) + \kappa\omega_m(\sigma_h^2 - \sigma_{sp}^2)/c)\\
r^e_{pp} &= -g^{-1}(\sigma_{pp}(2\kappa^2 + \kappa\omega_m\sigma_{ss}/c) + \kappa\omega_m(\sigma_h^2 - \sigma_{sp}^2)/c)\\
r^e_{ps} &= -2g^{-1}\kappa\omega_m(\sigma_{sp} - \sigma_h)/c\\
r^e_{sp} &= -2g^{-1}\kappa\omega_m(\sigma_{sp} + \sigma_h)/c\\
g &= (2\kappa + \omega_m\sigma_{ss}/c)(2\omega_m/c + \kappa\sigma_{pp}) + \kappa\omega_m(\sigma_h^2 - \sigma_{sp}^2)/c.
\end{aligned}$$

These additionally match the forms suggested by the Referee. The equations stating these reflection coefficients have been updated in the manuscript, with an additional comment on this issue following on lines 798-803.

We have corrected all of our calculations and numerical results to reflect this change. It is not trivial to see what consequence this would have on our quantitative results, but we are delighted to report that for our considered parameter ranges the rough trend is a reduction of energy scales by a factor of $\sim 1/2$. As of such, the interpretation of this new data follows

that of the previous dataset almost completely. We note that the slight shift in preferred stripe angle away from $\theta = 0$ due to the supposed symmetry breaking of the magnetic field, previously Appendix A, is no longer present after this change. We have confirmed that the Casimir contribution to the free energy is indeed actually invariant to time reversal symmetry, even though the system as a whole is not, per the previous Appendix A. We have correspondingly removed this discussion from the Manuscript.

> Moreover, formula (1), which gives the Casimir energy per unit area for two parallel planar surfaces, is used here to estimate a Casimir energy per stripe (or per particle). However, it is well known that the Casimir interaction is generally non-additive. For this reason, the authors should be more critical about the applicability of this approximation and clearly state under which conditions it is expected to be valid.
>
> In particular, the use of a planar energy density to estimate the energy of individual stripe domains is expected to be justified only when the lateral size of the stripe domains (typical values are not given in the manuscript) is large compared to the separation between the 2DES and the birefringent plate. In this regime, edge effects and non-additive contributions should be suppressed. Conversely, at larger separations, where the separation becomes comparable to or larger than the stripe domain size, the quantitative accuracy of the predicted energies is less clear. While the results at shorter distances may still be reliable, this limitation is not sufficiently discussed in the manuscript.

This is an important point regarding the approximation of translationally invariant 2DES properties. Ensuring that the typical stripe domain size exceeds the separation distance d is an underlying assumption of our work. If the disordered stripe domains are large compared to inter-plane distance d, we can approximate the system as a collection of non-interacting patches with well defined stripe direction, and the quantitative results are justified. The matter is complicated somewhat by the current lack of understanding of the stripe domain formation and size. In lieu of more accurate estimates, we can use the stripe ordering wavelength as a lower bound of this typical domain size —domains smaller than the wavelength of the density-wave-like phase are not conceivable. The stripe ordering length can be estimated from e.g. the self-consistent Hartree Fock theory to be $\Lambda \approx 2.8R_c$, see Ref. [45] of the revised Manuscript and References therein, where $R_c = l_B\sqrt{2N+1}$ is the cyclotron radius, $l_B = \sqrt{c\hbar/eB}$ the magnetic length, and N the number of fully occupied (spin degenerate) Landau levels. For typical parameters $\nu = 15/2$, $n = 2.9 \times 10^{11}$ cm^{-2}, and an effective electron mass of 0.067 m_e the ordering length is $\Lambda \approx 150$ nm, and it is reduced to $\Lambda \approx 100$ nm for the lowest relevant filling factor $\nu = 9/2$. As we find Casimir control viable below $d \approx 300$ nm, the mere stripe order wavelength is comparable to the interplane distance for large portions of the regime of interest. Considering that stripe domain sizes are most likely larger than the mere stripe ordering length, we conclude that our treatment of the stripe domains is appropriate in the entire experimentally viable window of d, and doubtlessly so towards the lower end of distances.

A discussion of this limitation has been added at lines 276-290.

> Minor comments:
>
> - Line 198: The quantities appearing in the expression for the filling factor (such as n and B) are not explicitly defined, although their meaning is rather standard. For clarity and completeness, these quantities should be briefly explained when they first appear.
>
> - Line 651: While most quantities entering the subsequent equation are reintroduced and explained, the anisotropy ratio λ is not defined again at this point. I suggest to restate its definition here for completeness and readability.

- In several parts of the Methods section, the speed of light c appears to be set to unity, while in the main text it is not. This can be seen, for example, when comparing the expression for κ following Eq. (1) with the expressions for the reflection matrix following line 628. See also the formulas following line 674.

We appreciate the finding of these typos, and have fixed these in the manuscript.

List of changes

Here follows a full list of changes made to the manuscript.

- An inconsistency in the polarization vectors used in deriving reflectivities has been corrected. The 2DES reflection coefficients following line 797 have correspondingly been changed, with additional comments added on lines 798-803.
- All data for all plots has been updated to reflect the new reflection coefficients. New plots have been produced with this data, with additionally a slight narrowing of the parameter ranges of filling factor ν and electron densities n displayed.
- The non-zero preferred twist angle was found an artifact of the previous reflection coefficients, and the related discussion has been removed from Fig. (2). and from lines 408-414, as well as the previous Appendix removed.
- A new Appendix detailing the spectral decomposition of the Casimir control has been added on lines 1118-1179, referenced on lines 334 and 664-667.
- A clarification on the scope of our own novel contribution has been added on lines 71-75.
- The typical stripe ordering wavelength is now mentioned on lines 142-144.
- Definitions of the parameters B, n, e, and $\hbar$ are now given on lines 208-210.
- The possible impact of dielectric screening on stripe microscopics is now discussed on lines 248-253.
- A discussion on the typical stripe domain sizes has been added to lines 276-290.
- The underlying assumptions of Equation (1) have been clarified on lines 306-323.
- The origin of the taken parameters τ, n, and ν is now mentioned on lines 396-399.
- The definition of the energy scale ΔF is now reiterated on lines 417-418 and in the caption of Figure (3).
- Lines 420-429 have been removed for a more conscise discussion.
- The stability of the results against experimental considerations is now discussed on lines 517-531.
- A claim which hinged on the precise working definition of “cavity” has been removed on lines 573-576.
- Recent work on LC circuits coupled to Casimir setups (Ref. 70), and on singling out effective modes from the free space background (Ref. 69), are now discussed on lines 604-615.
- A more in-depth discussion of dielectric screening of quantum Hall stripe microscopic theory has been added to lines 728-752.

- A footnote connecting our work to the Dicke phase transition has been added to line 64.
- The statement of stabilization up to 10^4 times larger than previously known stabilization energy scales has been adjusted to a factor 10^3 in light of the new data, adjusted on lines 22 and 509.
- The introduction now cites additional previous work on lines 41-45.
- New References [7-8], [11-15], [17-29], [58], [65], [69-70], [78-80] have been added and referred to in the manuscript.
- Minor edits, including correction of some typos, have been carried out to improve to text.

References

[1] G. Bressi et al. "Measurement of the Casimir Force between Parallel Metallic Surfaces". en. In: *Physical Review Letters* 88.4 (Jan. 2002), p. 041804. ISSN: 0031-9007, 1079-7114. DOI: 10.1103/PhysRevLett.88.041804. URL: https://link.aps.org/doi/10.1103/PhysRevLett.88.041804 (visited on 09/05/2025).

[2] F Intravaia and A Lambrecht. "Surface plasmon modes and the Casimir energy". In: *Physical review letters* 94.11 (2005), p. 110404.

[3] V. Adrian Parsegian. *Van der Waals Forces: A Handbook for Biologists, Chemists, Engineers, and Physicists*. en. Cambridge University Press, Nov. 2005. ISBN: 978-1-139-44416-3.

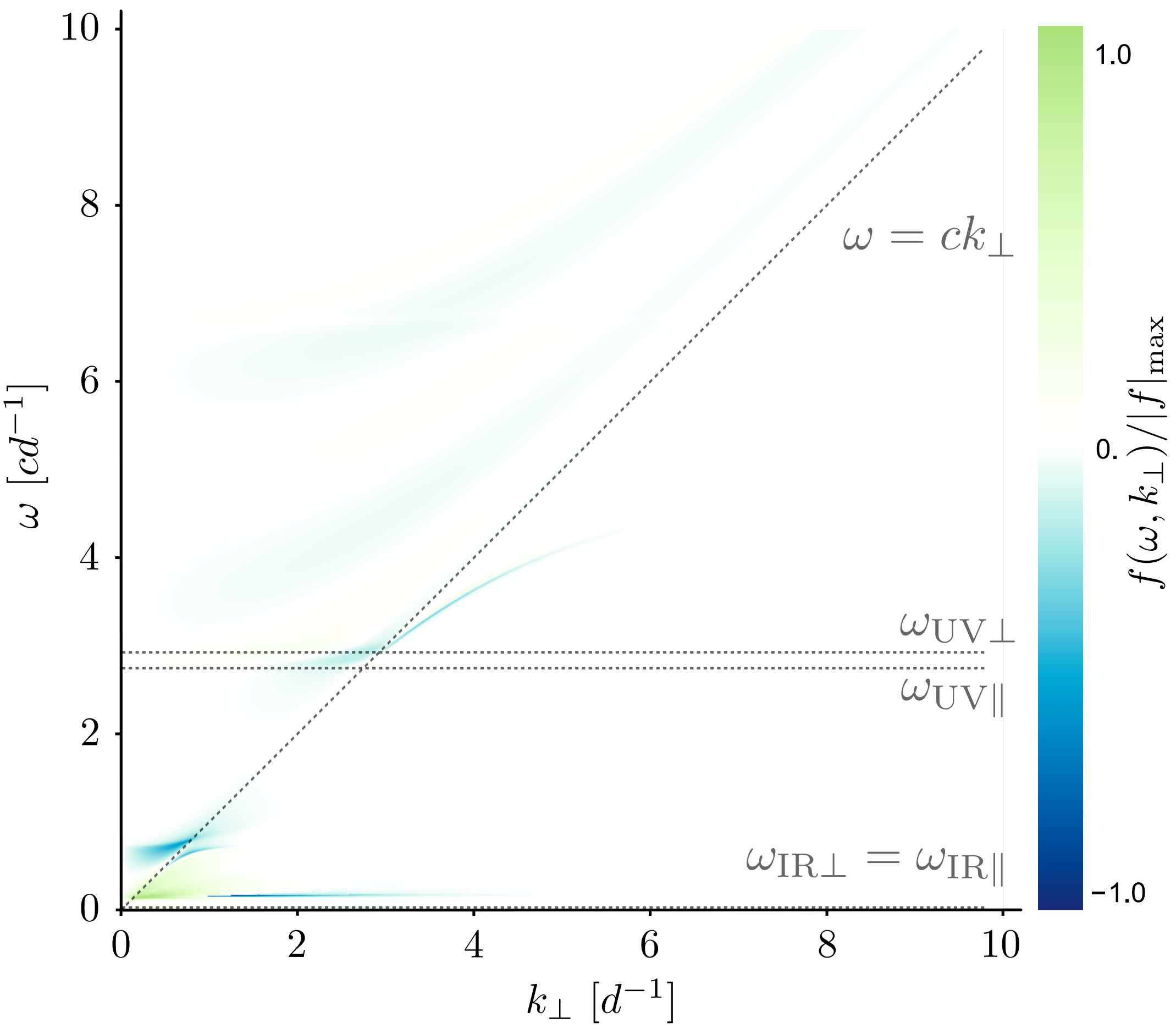

Figure 1: *Normalized real-frequency resolved Casimir integrand* $f(\omega, k_\perp)$. The values corresponding to twist angle $\theta = \pi/2$ have been subtracted from the preferred $\theta = 0$. This illustrates the competing contributions from various normal modes of the system to the energy scale ΔF. Density is here $n = 2.9 \times 10^{11}$ cm^{-2}, Drude scattering time is $\tau = 1$ ns, 2DES anisotropy is $\lambda = 55$, distance is $d = 100$ nm, and 2DES filling factor is $\nu = 15/2$. Birefringent plate oscillator frequencies $\omega_{\mathrm{IR}\perp}$, $\omega_{\mathrm{IR}\parallel}$, $\omega_{\mathrm{UV}\perp}$, $\omega_{\mathrm{UV}\parallel}$, have been overlaid. The characteristic frequencies of the 2DES Ω_c, τ^{-1}, lie far below $\omega_{IR\perp} = \omega_{IR\parallel}$, and have not been overlaid. Contributions from the right hand side of the free photon dispersion $\omega = ck_\perp$ are due to modes evanescent between the planes, the left hand side of the same corresponds to traveling modes. Only dependence on photon transverse momentum magnitude $k_\perp$ is shown, and contributions from different azimuthal directions ϕ have been integrated over.

June 2, 2026

Dear Referees,

Thank you once again for your thorough reading of our Manuscript. Here follow replies to the final comments left after the first round of reviews.

Yours,

Ola Carlsson, Sabuddha Chattopadhyay, Jonathan B. Curtis, Frieder Lindel, Lorenzo Graziotto, Jérôme Faist, and Eugene Demler.

Referee # 2

> Thank you for the detailed responses. All of my concerns have been addressed.

We would like to extend our thanks to the thorough reading of our Manuscript, and are happy that all concerns have now been treated appropriately.

Referee # 3

> Dear editor, dear authors,
>
> I would like to thank the authors for their detailed responses and the substantial effort put into revising the manuscript. They have addressed the vast majority of my previous concerns effectively, and I believe the manuscript is significantly stronger as a result.
>
> I have only a few remaining minor remarks and clarifications before recommending the manuscript for publication:
>
> 1. There are a couple of minor inconsistencies with equation citations. Following Equation (1), the text cites "Eq. (4)", which likely should refer to Equation (1) itself, as no Equation (4) exists in the manuscript. Equation (4) is also referenced in the paragraph before the methods section, in the first paragraph of the methods section, and right before the final form. Please double-check and correct these references.
>
> 2. There appears to be a minor dimensional issue regarding the reflection matrix for the 2DES. While the conductivity σ seems to be defined in SI units, the current expression for the reflection coefficient seems to assume it is dimensionless. It looks like the vacuum impedance (Z_0) might be missing from the reflection formula. Furthermore, to avoid any ambiguity, it would be worthwhile to explicitly state in the methods section that the SI unit system is chosen.
>
> Once these final minor points are integrated, I am fully in support of the publication of this work.

We thank the Referee for the kind words, and for finding these typos in the Manuscript. These have corrected them in the new revision.

Indeed the new section on real frequency integrand evaluation accidentally used the same LaTeX label for its equation as that of Eq. (1), causing the first issue.

Indeed the vacuum impedance had been set to one in most of our calculations, being reintroduced first on the level of the numerics. We have adjusted the equations detailing the reflection properties of the 2DES to align with the SI units used elsewhere, using the relation $Z_0 = 1/\varepsilon_0 c$. We note that the usage of SI units is mentioned, if briefly, in the third paragraph of the Methods section.

Further changes

We have in addition to the changes noted above adjusted the Manuscript to conform better to *Nature Communications* formatting standards. As of such, the abstract has been condensed, as has been removed the usage of footnotes.

The theoretical manuscript proposes controlling the orientation of fluctuating nematic order in the quantum Hall stripe phase of a 2D electron system (2DES) by placing it near a birefringent $BaTiO_3$ plate and exploiting an orientation-dependent Casimir (vacuum) free energy. The Casimir free-energy contribution is computed within a scattering-formalism framework from Ref. [35] and [36] , using reflection matrices constructed from a dielectric tensor for $BaTiO_3$ and a modified Drude-type conductivity tensor for the anisotropic (stripe) 2DES. The manuscript then studies how the resulting stabilization energy varies with twist angle and with the separation between the 2DES and the birefringent plate, and argues that this energy scale can align otherwise fluctuating stripe domains, yielding a preferred macroscopic nematic orientation.

The central idea is interesting, and the calculations appear internally consistent within the stated model; however, several key claims (especially regarding experimental robustness, physical interpretation, and positioning in the broader cavity–vacuum-control literature) require clearer justification and/or additional analysis before the conclusions can be considered convincing.

Major comments

Comment #1: The manuscript would benefit from a clearer statement of the main conceptual/technical contribution. The scattering-theory approach to Casimir free energy is not new (Ref. [35] and [36]), and the present work appears to apply a known formalism by inserting specific material response functions ($BaTiO_3$ dielectric tensor and stripe-state conductivity) to obtain an angle-dependent free energy; the authors should articulate explicitly what is new beyond this application (e.g., a new mechanism, a new scaling regime, or a new experimentally relevant estimate).

Comment #2: The physical mechanism is presented mainly in terms of the scattering formula for the free energy, but the manuscript does not clearly explain how the electromagnetic normal modes (or, equivalently, the photonic density of states) change with twist angle and how that translates into the observed angular dependence of the free energy. A qualitative explanation (even schematic) connecting twist angle, mode structure, to free-energy anisotropy would greatly improve the manuscript.

Comment #3: It is unclear whether (and how) dielectric screening and electrostatics associated with the nearby plate/substrate are treated consistently. In a real device, a nearby dielectric modifies Coulomb interactions in the 2DES and can shift energetics of stripe formation itself; the manuscript should clarify whether the “control plate” is assumed to only affect the electromagnetic vacuum fluctuations or also modifies electron–electron interactions through static/dynamic screening, and what the expected magnitude of that effect is.

Comment #4: Figure 3 reports a stabilization energy vs separation d and implicitly implies strong distance dependence. If the Casimir free energy changes significantly with d, there is also an associated normal Casimir force between the bodies; the manuscript should estimate the magnitude of this force (or force per area) for the parameter regime used and comment on whether such forces are mechanically reasonable/compatible with typical experimental

constraints, ideally with at least one comparison to measured Casimir-force scales in related geometries.

Comment #5: The manuscript emphasizes that single/few-mode cavity-QED pictures are insufficient and that continuum-mode Casimir physics is essential. However, no spectral decomposition is provided to show which frequency and in-plane momentum ranges dominate the stabilization energy (or the difference in free energy between orientations), so the continuum nature remains asserted rather than demonstrated. It would be nice to add quantitative analysis/comparison to clarify this point.

Comment #6: The introduction states broadly that vacuum-field cavity control has been explored across ferroelectricity, superconductivity, and ferromagnetism, but several important and widely cited contributions from other groups are missing. At minimum, the literature overview should be expanded so that the manuscript does not read selective; the authors should cite representative works from multiple communities (electron–photon coupling in solids, phonon-polaritonic/ferroelectric routes, cavity-modified superconductivity, cavity-magnetism proposals), and clarify how their "Casimir control" differs from those approaches.

Comment #7: Relatedly, additional review articles on cavity control of materials (beyond the single review currently cited) should be included, and the introduction should be revised to better reflect the breadth of the field and avoid giving the impression that only a narrow set of early proposals exist. The manuscript would benefit from a short paragraph that explicitly distinguishes "Casimir control" (renormalized, mode-continuum, non-resonant, geometry/material engineered) from more standard cavity-QED-inspired approaches.

Comment #8: The manuscript's framing would be strengthened by engaging with recent work in Fabry–Pérot-like cavity settings showing that, in some regimes, a continuum of photon modes can be approximated by one or a few effective modes while still removing free-space contributions through appropriate renormalization. This is directly relevant to the manuscript's claim that single/few-mode descriptions are generically insufficient, and the authors should clarify the scope/limits of that claim.

Comment #9: The key quantitative claim is that the orientation-dependent Casimir free energy can reach "stabilization energy scales up to 10^4 times larger than previously known mechanisms" for stripe alignment. This claim is extremely strong, yet the manuscript does not provide a sufficiently careful uncertainty analysis (dielectric function model choices, heterostructure layering, losses, finite thickness) to justify it as a general or experimentally robust statement.

Minor comments

Comment #10: When introducing Eq. (1) (the scattering-formalism expression for the free energy), it would be helpful to briefly state the key assumptions and limitations (e.g., planar geometry, linear response, locality/nonlocality assumptions, how material dispersion and losses enter, and what is meant by the renormalization/subtraction).

Comment #11: In Fig. 3, the manuscript should explicitly state which twist-angle configuration is used to define the plotted stabilization energy, so that the plotted quantity is unambiguous.

Comment #12: Please justify (briefly) the specific parameter choices used in the main figures (e.g., d, nu, tau, and n) and clarify whether they are representative of particular experiments or chosen for numerical convenience.

Comment #13: On page 6, the authors mentioned that "Indeed, by considering this geometry, we broaden the scope of electromagnetic vacuum control outside of the commonly considered vacuum cavity regime." It seems that the control is still within the scope of cavity control, if we define a cavity as a device that confines electromagnetic vacuum within a confined space such as plaritonic devices and hBN phonon-polaritonic devices.

Comment #14: The acronym "2DES" appears before being defined (it is used on page 1 but defined later); please define it at first use for readability.
